# Movement Analysis Could Help in the Assessment of Chronic Low Back Pain Patients: Results from a Preliminary Explorative Study

**DOI:** 10.3390/ijerph19159033

**Published:** 2022-07-25

**Authors:** Stefano Negrini, Joel Pollet, Giorgia Ranica, Sabrina Donzelli, Massimiliano Vanossi, Barbara Piovanelli, Cinzia Amici, Riccardo Buraschi

**Affiliations:** 1 Department of Biomedical, Surgical and Dental Sciences, University of Milan “La Statale”, 20122 Milan, Italy; stefano.negrini@unimi.it; 2 IRCCS (Istituto Ortopedico Galeazzi), 20161 Milan, Italy; 3 IRCCS (Fondazione Don Carlo Gnocchi), 20148 Milan, Italy; granica@dongnocchi.it (G.R.); rburaschi@dongnocchi.it (R.B.); 4 ISICO (Italian Scientific Spine Institute), 20141 Milan, Italy; sabrina.donzelli@isico.it (S.D.); massimiliano.vanossi@isico.it (M.V.); 5 Physiotherapy Clinic, 89069 San Lorenzo, Italy; barbarapiovanellift@gmail.com; 6 Department of Mechanical and Industrial Engineering, University of Brescia, 25123 Brescia, Italy; cinzia.amici@unibs.it

**Keywords:** chronic low back pain, spine, movement analysis, patient outcome assessment, movement

## Abstract

**Introduction**: This study aimed to assess the reliability of a qualitative scoring system based on the movement analysis of the spine in different populations and after usual care rehabilitative intervention. If proven true, the results could further future research development in quantitative indexes, leading to a possible subclassification of chronic low back pain (cLBP). **Methods**: This was a preliminary exploratory observational study. Data of an optoelectronic spine movement analysis from a pathological population (cLBP population, 5 male, 5 female, age 58 ± 16 years) were compared to young healthy participants (5M, 5F, age 22 ± 1) and were analysed via a new qualitative score of the pattern of movement. Internal consistency was calculated. Two independent assessors (experienced and inexperienced) assessed the blinded data, and we calculated inter- and intrarater reliability. We performed an analysis for cLBP pre and post a ten session group rehabilitation program between and within groups. **Results**: Internal consistency was good for all movements (α = 0.84–0.88). Intra-rater reliability (Intraclass correlation coefficient–ICC) was excellent for overall scores of all movements (ICC_(1,k)_ = 0.95–0.99), while inter-rater reliability was poor to moderate (ICC_(1,k)_ = 0.39–0.78). We found a significant difference in the total movement scores between cLBP and healthy participants (*p* = 0.001). Within-group comparison (cLBP) showed no significant difference in the total movement score in pre and post-treatment. **Conclusion**: The perception of differences between normal and pathological movements has been confirmed through the proposed scoring system, which proved to be able to distinguish different populations. This study has many limitations, but these results show that movement analysis could be a useful tool and open the door to quantifying the identified parameters through future studies.

## 1. Introduction

Low Back Pain (LBP) is one of the leading causes of disability worldwide [1]. When the condition becomes chronic (cLBP), the association of many different factors drives the consideration of LBP as a bio-psycho-social condition. In cLBP, the main treatment focus is to reduce disability, however, there is insufficient evidence about effective interventions to reduce and possibly solve the symptoms [2]. Moreover, many common interventions such as education, or manual and electrical modalities have poor or no efficacy [2,3,4,5]. In the rehabilitation community, there is consensus that a detailed evaluation should guide the treatment choices to provide cLBP patients with the most effective individualized approach, even if there is no agreement on what this evaluation should include [2]. While imaging is mostly prescribed in the post-acute phase to rule out any secondary cause [6], in cLBP clinical expert assessment it is proposed as a possible solution to individually drive therapeutic choices. When considering imaging in the assessment of LBP, and in particular, cLBP patients, it is important not to be misled by the results of the imaging. It is known that alterations of the spine are also common in healthy subjects and in most cases not related to the patient’s symptoms [2]. Research has shown that patients can be subclassified according to the clinical presentation to allow personalized approaches, demonstrating good results [7,8]. Biomechanics could play an important role among the biological factors of the bio-psycho-social cLBP syndrome.

Movement analysis, particularly kinetic and kinematic assessment, is widely adopted in rehabilitation as an important instrument to obtain objective, repeatable, and sharable measurements to complete a clinical assessment [9]. Gait analysis is considered the gold standard to measure functional deficit, supporting decision making and providing follow-up analysis for functional surgery, botulin toxin injection, and rehabilitation interventions in cerebral palsy management. It is used in stroke survivors, amputee patients, and in the orthopaedic field for hip and knee arthroplasty. Additionally, for the upper limbs, movement analysis is used to study kinematics and pathological patterns in cerebral palsy, obstetrical brachial plexus palsy, and stroke rehabilitation participants. An application of this technology to trunk movements has been performed through stereophotogrammetry [10], infrared surface topography [11], or wearable sensors [12,13]. The analysis of the trunk through optoelectronic systems has been performed scarcely [14], with only a few exceptions [15]. Implementing this technology can spur new interesting insights into the biological factors influencing cLBP [16].

From the previous literature, we know that cLBP patients have neuromotor answers to specific external stimuli that are different from normal ones, such as reflex inhibition [17], changes in anticipatory stabilization [18], absent flexion, and relaxation phenomenon [19], and altered motor answers to external stressors [20]. Moreover, in our everyday clinical experience, we have seen some gross movement alteration during the classical range of motion exploration. Nevertheless, these cannot be studied or reported without a movement analysis instrument to allow their quantification and classification.

We recently developed a trunk movement analysis which requires considerable effort and time to measure patients’ data. The automatic quantitative analysis of movement quality has proven particularly challenging and deciding what to evaluate and how has been difficult. We found it essential to perform a preliminarily study, where possible, to elucidate some of the qualitative characteristics that are reliable and sensitive to changes, and which differentiate normal movements from pathological movements before embarking on a more complicated numerical analysis. Consequently, we developed a study to assess the reliability of a preliminary qualitative scoring system of spine movements. This score will serve mainly as a first preliminary classification to address future quantitative research efforts. We do not expect to use this score in clinics. In our mind, it is a research instrument to identify which movement patterns should be prioritized in the subsequent engineerization for quantitative purposes. If this latter phase fails, we could use the score after more extended and appropriate research. For this reason, we explored and studied different aspects and features of the score: reliability, discriminatory properties between a pathological population (cLBP patients) and healthy young participants, sensitivity to change in the standard of care, and correlations with pain and disability after rehabilitation. The rehabilitative intervention proposed was a usual care program administered to the cLBP population administered at our centre. This exploratory study will identify new directions for research and guide the choice of the parameters to be quantified in the near future.

## 2. Methods

### 2.1. Design and Setting

This is a preliminary exploratory prospective case-control validation study conducted in the motion analysis laboratory of our centre from March 2018 to November 2019. In this study, we aimed to check if it was possible to identify specific repeatable pathological movement patterns, if these were able to distinguish different populations, and if these changed with movement-based usual care exercise therapy. The study was conducted according to the Declaration of Helsinki and approved by the local Ethical Committee.

### 2.2. Participants

After receiving written informed consent, we recruited 10 participants diagnosed with cLBP. The inclusion criteria were having cLBP for at least 6 months and being between 18 and 70 years (both sexes). Exclusion criteria were having had previous spine surgery, inflammatory disorders, and rheumatic diseases. We randomly extracted a healthy sample to represent the “best possible” movement pattern from our overall population database. For this reason, we extracted the data of 10 young participants according to the following criteria: aged between 18 and 30 years, both sexes, no history of low back pain in the last 12 months, no orthopaedic diseases at the spine, and no inflammatory disorders. A clinician specialist in physical medicine and rehabilitation was responsible for the cLBP patient recruitment.

### 2.3. Evaluation Protocol

**Figure 1:**
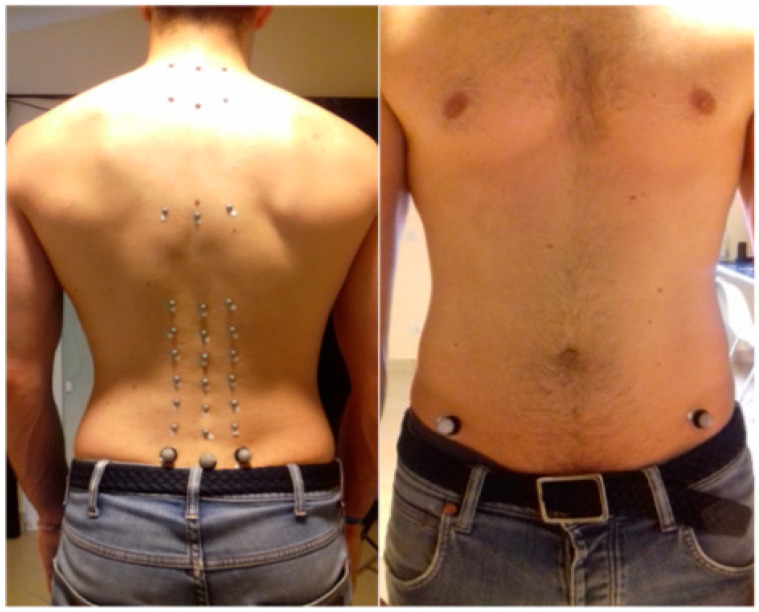
Positioning of the 32 markers.

For the movement analysis, we asked participants to remove their shirts, shoes, and clothing accessories. On each subject, 32 markers in specific anatomic landmarks (Figure 1) were placed by two expert physiotherapists [EF, RN]: (1) on spinous and both transverse processes of C7, T3, T7, T12, L1, L2, L3, L4, and L5, (2) on the spinous process of S2, and (3) bilaterally on ASIS and PSIS. All markers had a diameter of 5 mm except for those of 10 mm on S2, ASIS, and PSIS. Repeatability of marker placement has been preliminarily studied (intra-operator error of 4.10 ± 0.49 mm, and inter-rater error of 1.3 ± 1.9 mm). To record the spatial components of the 3D displacement of the markers during the subject’s movements, we used a BTS SMART-DX 400 (BTS Bioengineering, Garbagnate, Italy), with eight cameras, with an acquisition frequency of 100 Hz. The position of the cameras was adjusted according to the height of each subject (at least 20 cm above the participants’ heads). The 3 repetitions were separated and each one started with a ‘go’ signal provided by the same operator. All movements were performed standing, with no instruction given about the foot’s position. Each subject was asked to perform at a self-selected speed and to go as wide as possible in the following movements 3 times: trunk flexion and return, right and left lateral bending, and right and left rotation. All the movements were performed three times as a warmup before the examination, according to specific instructions: for the flexion movement, to bend forward on the sagittal plane, for the lateral bending, to bend laterally by sliding the upper limb along the ipsilateral lower limb, while for the rotation, to hold the lower limbs as steady as possible while rotating and looking to the right and the left. The duration of the entire evaluation was about 30 min. The tests were performed between 8 and 12 am. We did not test the lumbar extension movement because it covered the markers placed on the back with the current camera setup. Consequently, in this study, we focused only on the other movements.

Healthy participants and cLBP patients followed the same procedure for data collection. Evaluations of cLBP patients were conducted twice, at T0 (the first day of rehabilitation intervention, before the session) and T1 (the last day of rehabilitation, after the session). Before each acquisition, we also administered the Numeric Pain Rating Scale (NPRS) [21] and the Oswestry Disability Index (ODI) [22].

### 2.4. Usual Care Exercise Therapy Intervention

The cLBP participants attended a 10-session group rehabilitation program, for two weeks with five sessions per week for 30 min each session. A physiotherapist with 12 years of experience led the intervention. The outpatient program consisted of exercises for strengthening trunk and hip muscles, self-spine mobilizations, core-stability exercises, active stretching, breathing techniques, relaxing exercises, and postural re-education. The program did not include home exercises but suggested implementing and increasing daily physical activities.

### 2.5. Data Analysis

**Figure 2:**
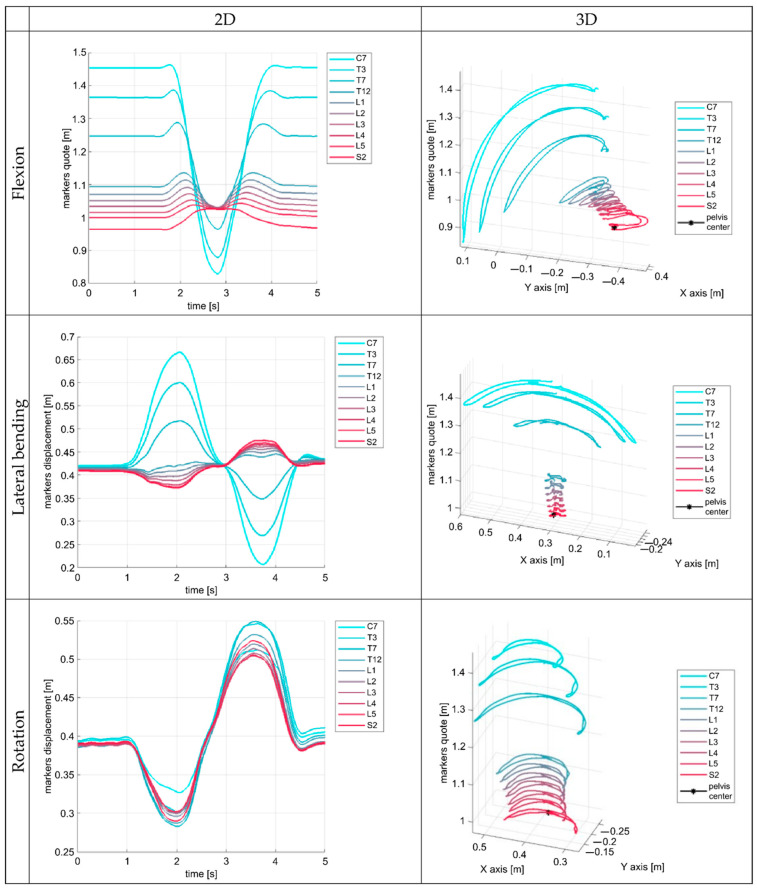
Plots of marker’s displacement and Z component with respect to time of a healthy subject during movement analysis. In a qualitative analysis, these graphs repeat constantly in the healthy population with few variations [23]. Each line represents the displacement in time of a triplet of markers placed on the spinous and transverse processes of individual vertebrae (see text).

The acquired data were blinded, imported into the MATLAB^®^ (MathWorks^®^ The MathWorks, Inc., Natick, MA, USA) environment, and elaborated through a specifically developed custom code. For the analysis, we used a cartesian reference frame, defined as follows: (1) generated by the Z direction, (2) Z direction orthogonal to the ground with a positive orientation from the ground to the subject’s trunk, (3) X direction aligned to the subject’s sagittal plane, with a positive orientation from the subject’s back to the front, and (4) Y direction and orientation consequently defined. For each marker, we considered the three-dimensional displacement raw components X, Y, and Z, and the absolute displacement *R* was evaluated as a vectorial sum of the spatial components. Furthermore, for each subject and trial, an anthropometric factor *h* was computed as the mean distance between the quote of spinous C7 marker and spinous S2 marker in the first 10 seconds of recorded data, corresponding to a resting position for the subject. The quote was defined as the distance to the horizontal plane. The pelvis centre was evaluated as the mean value of the ASIS and PSIS markers position during the first second of acquisition. For the operators’ evaluation, two plots were generated from each acquisition, depicting: (1) the markers’ spatial displacement (3D), and (2) the markers’ Z component with respect to time (2D) (Figure 2). An univocal code was assigned to each pair of plots by a researcher not involved in the plot evaluation (CA) to blind the graphs.

During a previous study [23], through repeated and careful observations of the movement graphs of fifty-five healthy participants, some movement characteristics were identified. This observation allowed us (SN, BP, and CA) to identify some qualitative features for each movement in healthy participants (Table 1). For the flexion and extension task, the first attempt at quantitative assessment of the most relevant parameters and alternative descriptions of those quantities can be found in [24].

### 2.6. Evaluation

We designed a quality visual scoring system using a 4-point Likert-type scale to rate the graphs. Each movement feature ranges from 0, the better score (typical smooth movement of a healthy subject), to 3, the worst score (very far from a typical healthy movement). We computed an overall score for each movement (the sum of features scores) and a total movement score (the sum of each overall score). The full description of the scoring criteria is reported in Appendix A. All the items identified through the preliminary analysis have been analysed and included in the study.

Two raters blindly evaluated each graph: one had four years of experience in movement analysis (BP), and the other was inexperienced (JP). The inexperienced observer performed one assessment (for a total of 540 graphs assessed), whereas the expert observer performed two assessments at a one-month interval one from the other (for a total of 1080 graphs assessed). Each observer was blinded to the subject evaluated and to the other one’s results, and they were not allowed to discuss the single assessments. The experienced rater contributed to developing the scoring criteria, while the other received a 2 h training. The raters were allowed to consult the scoring criteria explanation (Appendix A) during the scoring process.

### 2.7. Statistics

We provide a descriptive analysis of the sample with mean and standard deviation for continuous variables and proportion for categorical variables. We calculated differences in the baseline parameters between healthy and cLBP participants with a Student T-test. To assess the internal consistency of each set of measurements (flexion and return, lateral bending, and rotation) Cronbach alpha was calculated, and the interitem covariance and the average covariance were checked and reported. We calculated the one-way random effect intraclass correlation (ICC) with single rater measurements for the overall scores obtained on the same patient by the same assessor to check the intra-rater reliability. We utilized a one-way random effect ICC with the mean of *k* raters/measurements of the same patient by two independent assessors to assess the inter-rater reliability. The ICC absolute agreement is reported with a 95% confidence interval. For the ICC interpretation, the following cut-offs were used: 1.00 to 0.75 excellent, 0.74 to 0.60 good, 0.59 to 0.40 fair, and 0.39 or less poor.

We checked differences between healthy and cLBP participants (at baseline and post-intervention): due to the non-Gaussian distribution of data, we ran a Kruskal Wallis test with a Neuman–Keuls post hoc analysis. We performed the Mann Whitney U test for between-groups analysis, and Wilcoxon Sign rank test for differences between baseline and follow-up in cLBP participants for total movement score, NRS, and ODI scores.

## 3. Results

Patients were older, and with a higher BMI than healthy participants (Table 2).

Internal consistency was good for all movements (Table 3). The ICC (1,k) was good to excellent for flexion and lateral bending, whereas rotation showed poor reliability in comparing operators with different expertise (Table 4). The intra-rater reliability was excellent for the overall score of all movements but slightly better for flexion and lateral bending.

**Figure 3:**
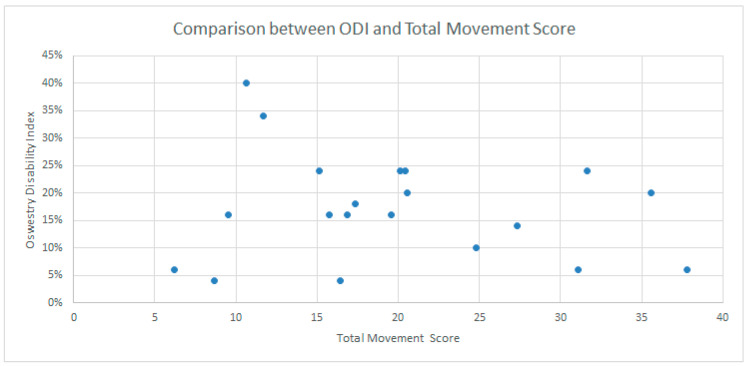
The plot of the values of disability (ODI%) and the total movement score values.

We found a statistically significant difference between healthy and participants with cLBP for the total movement score (*p* = 0.016). The difference was significant in the comparison between healthy and cLBP participants at baseline, with a mean difference (delta) of the total movement score of 5.77 *p* = 0.002, and between the end of follow-up and healthy participants with a mean difference (delta) of 5.00 and *p* = 0.008 (Table 5). We found no significant changes in the score for the ten patients with cLBP (Table 6). The correlation graph between the total movement score and ODI score showed a “cloud shape” (Figure 3).

## 4. Discussion

In this exploratory study, we found that spine movement analysis allowed the identification of reliable specific qualitative movement patterns. We also found that these patterns were able to distinguish between populations, even if we could not determine whether this was due to age or pain. Conversely, usual care exercise therapy did not change the total movement score. These results allowed the total movement score to progress further in engineering and its elements to see if a more accurate quantification is possible. The current total movement scores could also be investigated further, looking for definitions of the intervention and comparisons more accurately than in the current study.

The rater’s experience should be taken into account: while the intra-rater reliability was excellent [25], the inter-rater (experienced and inexperienced) reliability was moderate to good for flexion and lateral bending but poor for rotation. Further studies should compare raters with the same experience to assess their reliability. Moreover, the issue of inter-rater reliability could be solved with the definition of specific cut-offs for each scoring criteria. This could lead to a more objective evaluation and improvements in the inter-rater reliability. However, this improvement process needs the availability of a large amount of normative data and a subsequent quantitative analysis of the trunk movement.

The relevance of the method as a follow-up evaluation or as an outcome measurement tool remains uncertain. We applied the movement analysis and the scoring system to a real-life outpatients rehabilitation practice [26], where the intervention for cLBP was given in heterogeneous groups with a delayed time from prescription to administration, up to 1 month on a waiting list, and with an intensity of 10 sessions over 2 weeks. In this context, our cLBP sample did not show changes in the outcomes from pre to post-treatment [27]. This was consistent with the obtained movement score that likewise did not change from baseline to follow-up, but it did not allow us to evaluate any correlation between outcome measurements and movement scores.

The choice of the analysed movements has a clinical and technical explanation. From a clinical point of view, the movements are among the typical ones tested by clinicians during physical examination. On the technical side, those movements allow the markers to be always visible during the test by the system’s cameras, which does not happen, for instance, during an extension movement.

This study represents a novelty in the field of LBP because it presents a new possible analysis of the problem focusing on the functional impairment caused by LBP, instead of the biological or mechanical lesion [28], which better fits the current vision of LBP as a bio-psycho-social condition [29,30]. Future studies should consider a quantitative approach to achieve a less operator-dependent procedure, and the application of the scoring system to a sample undergoing a rehabilitative intervention of proven effectiveness.

This study has several limitations beyond the already cited problems with a qualitative evaluation of movement, such as the limited sample of cLBP patients included in the study. However, the novelty represented by this study and the design of this preliminary exploratory study could justify the small sample size. The complexity and time-consuming procedures for data collection and analysis necessitated a preliminary study before moving to a broader sample and improved analysis. A technical limitation is represented by the impossibility to detect the markers during the extension movement due to being covered by the shape of the paravertebral muscles. A relevant limitation is the wide range of ages in the cLBP group, and the difference in age, height, and weight between the two populations. There are some significant reasons for this. It is common in movement analysis to have a young reference sample serving as the phenomenon’s best physiological description. Moreover, the difference in age of the two studied populations, the use of a standard of care approach instead of treatment proven effective by previous studies, and the inclusion of patients with possible confounders such as previous treatments (even if no patient had had surgery previously or any other therapy in the previous six months) have a common explanation. Our aim was not (yet) to describe cLBP (or aging) or movement patterns or their specific changes in this study. We wanted to investigate whether it was possible to find any differences in the movement patterns of different populations and any changes in time, irrespective of the treatment. The next stages of this project will try to resolve all the limitations raised from this preliminary study by implementing a quantitative analysis. In this analysis, we will consider the time course of trajectories together with those of their derivatives. This could provide helpful quantitative insights to assess the possible presence of abrupt irregularities or intermittence automatically.

## 5. Conclusions

The perception of differences between normal and pathological movements has been confirmed through the proposed scoring system, which proved to be able to distinguish different populations. This preliminary exploratory study has many limitations, but these results show that movement analysis could be a useful tool and open the door to quantifying the identified parameters through future studies.

## Figures and Tables

**Table 1:** Movement features identified through the observation of 55 healthy participants (the scoring criteria for each feature is detailed in Appendix A).

Flexion and Extension	
**Feature**	**Description**
Start	The presence of a starting overshoot, as a detectable peak in the marker trajectory (opposite to the downward trend of flexion) before the profiles begin to decrease.
Fluency	The shape of the time trajectory is configured without sudden stops, jumps, or discontinuities. This is when the execution of the measured movement is fluid both in its trajectory and in speed and acceleration. From an analytical point of view, the graphs show a continuous trend of the trajectory and its temporal derivatives that express the continuity of velocity and acceleration during the execution of the motor task and therefore the absence of sudden irregularities or intermittence in the movement.
Total displacement/ Range of Motion	The amount of displacement retrieved in the graph.
End	The quote of the profiles at the end of the movement with respect to the starting point quotes.
Symmetry	How many descending and ascending parts of the profile represent symmetric slopes with respect to the ideal vertical straight line crossing the profile at the maximum flexion position.
Pelvis centre position	The position of the pelvis centre with respect to S2 at the maximal flexion point.
**Lateral Bending**	
**Feature**	**Description**
Fluency	The shape of the time trajectory is configured without sudden stops, jumps, or discontinuities. This is when the execution of the measured movement is fluid both in its trajectory and in speed and acceleration. From an analytical point of view, the graphs show a continuous trend of the trajectory and its temporal derivatives that express the continuity of velocity and acceleration during the execution of the motor task and therefore the absence of sudden irregularities or intermittence in the movement.
Total displacement/ Range of Motion	The amount of displacement retrieved in the graph.
Symmetry	The amount of overlap between the descending and ascending part of each curve (right and left) in the graph.
Cervico-thoracic/lumbosacral reverse movement	The opposite trend trajectory of C7 and S2 lines during movement.
Pelvis centre position	The position of the pelvis with respect to the midpoint of each line of the graph.
**Rotation**	
**Feature**	**Description**
Fluency	The shape of the time trajectory is configured without sudden stops, jumps, or discontinuities. This is when the execution of the measured movement is fluid both in its trajectory and in speed and acceleration. From an analytical point of view, the graphs show a continuous trend of the trajectory and its temporal derivatives that express the continuity of velocity and acceleration during the execution of the motor task and therefore the absence of sudden irregularities or intermittence in the movement
Total displacement/ Range of motion	The total amount of displacement is retrieved in the graph.
Symmetry	The amount of overlap between the descending and ascending part of each curve (right and left) in the graph.
End	The end height of the lines of the graph with respect to the starting point height.
Pelvis centre position	The position of the pelvis with respect to the midpoint of each line of the graph.

**Table 2:** Sample description.

	cLBP	Healthy	*p*
Gender	5F; 5M	5F; 5M	-
Age (year)	58 ± 16	22 ± 1	0.00
Height (cm)	168 ± 7	173 ± 6	0.20
Weight (kg)	75 ± 14	68 ± 8	0.20
BMI (kg/m^2^)	26 ± 4	23 ± 2	0.03
NRS (baseline)	5.5 ± 3	//	
ODI (baseline)	19 ± 8	//	

F = female; M = male; BMI = Body Mass Index; NRS = Numeric Rating Scale; ODI = Oswestry Disability Index; // = not applicabile.

**Table 3:** Internal consistency (Chronbach’s Alpha).

Movement	Average Interitem Covariance	Chronbach’s Alpha	Ranges
Flexion and return	0.60	0.88	0.10–0.80
Lateral bending	0.61	0.88	0.21–0.80
Rotation	0.45	0.84	0.20–0.81

**Table 4:** Intra-rater and inter-rater reliability.

			ICC	95%CI
**IC**	Intra-rater	Flexion and return	0.99	0.98–1.00
		Lateral bending	0.97	0.94–0.99
		Rotation	0.95	0.88–0.98
	Inter-rater	Flexion and return	0.72	0.36–0.89
		Lateral bending	0.78	0.53–0.91
		Rotation	0.39	−0.09–0.73

**Table 5:** Movement scores (overall and total) of participants with cLBP and healthy participants.

	cLBP T0	cLBP T1	Healthy
	Mean ± SD	Mean ± SD	Mean ± SD
Overall flexion score	6.3 ± 3.5	5.7 ± 1.9	2.8 ± 1.3
Overall lateral bending score	5.5 ± 2.9	5.4 ± 1.9	5.2 ± 1.4
Overall rotation score	6.4 ± 1.2	6.3 ± 1.9	4.3 ± 1.0
Total movement score	18.2 ± 6.3 *	17.4 ± 5.3 ^+^	12.4 ± 1.9

* Significant difference between the total movement score of participants with cLBP at T0 and healthy participants (*p* = 0.002); ^+^ Significant difference between the total movement score of participants with cLBP at T1 and healthy participants (*p* = 0.008).

**Table 6:** Average results (T0 vs. T1) of patients with cLBP differences that were tested with the Wilcoxon sign rank test.

Measures	Baseline (T0) Mean (SD)	Follow-Up (T1) Mean (SD)	*p*-Value
Total movement score	18.52 (7.74)	21.2 (10.40)	0.38
ODI (%)	19 (8.48)	16 (11.06)	0.27
NRS	5.5 (2.99)	3.2 (2.04)	0.07

## Data Availability

The data presented in this study are available on request from the corresponding author.

## Appendix A. Scoring Criteria

**Table A1:** Flexion and return features.

Feature	Scoring Visual Guide			
**Start**	**Score 0** = before the graph begins to decrease there is a clear opposite movement that creates a peak.			
	**Score 0**	**Score 1**	**Score 2**	**Score 3**

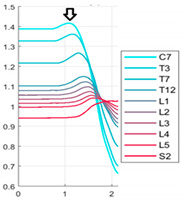
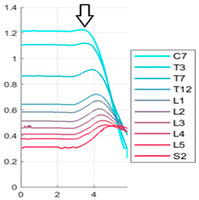
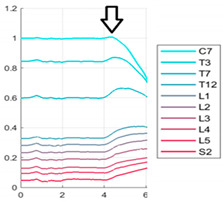
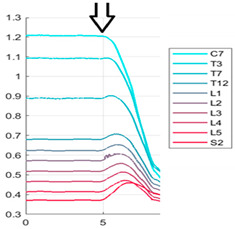
			
**Fluency**	**Score 0** = the graph lines are continuous with no intermittences.			
	**Score 0**	**Score 1**	**Score 2**	**Score 3**

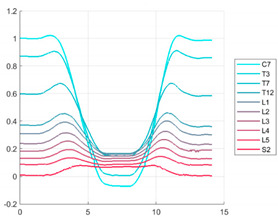
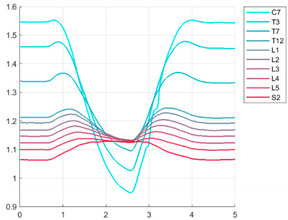
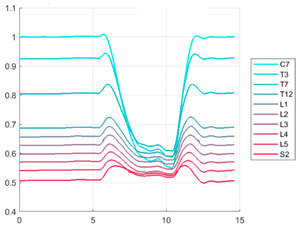
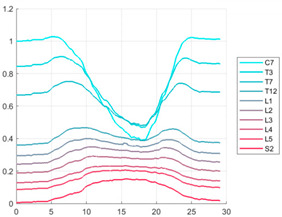
			
**Total** **Displacement**/ **Range** **of Motion**	**Score 0** = C7 trajectory line always crosses the S2 trajectory line (the more the C7 trajectory line crosses the S2 trajectory line, the better the ROM is).			
	**Score 0**	**Score 1**	**Score 2**	**Score 3**

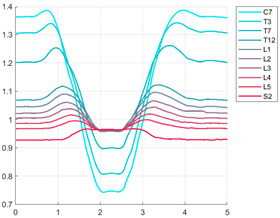
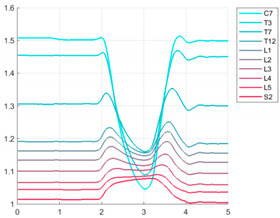
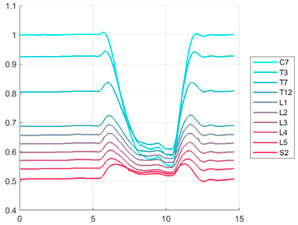
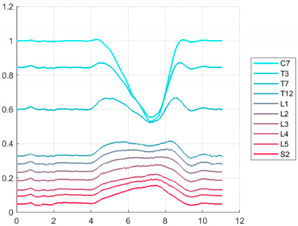
			
	**Score 0** = when the movement ends, the line of the graph is always at a higher level with respect to the starting line level.			
	**Score 0**	**Score 1**	**Score 2**	**Score 3**

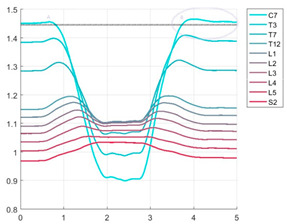
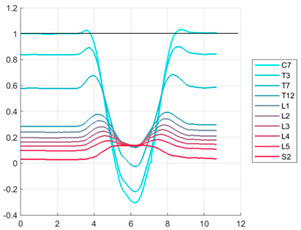
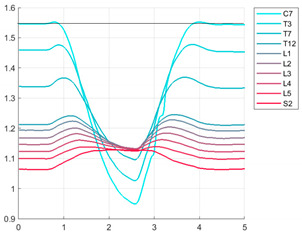
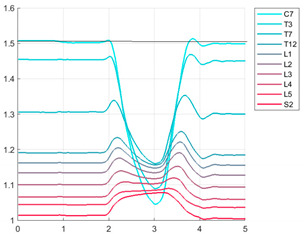
			
**Symmetry**	**Score 0** = the descending part of the graph overlaps with the ascending part.			
	**Score 0**	**Score 1**	**Score 2**	**Score 3**

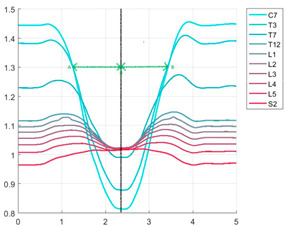
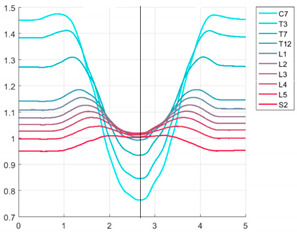
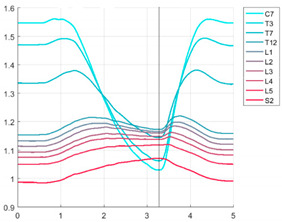
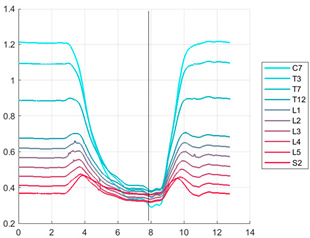
			
**Pelvis Centre Position**	**Score 0** = the pelvis centre should be in the point of maximal flexion of the markers identifying S2.			
	**Score 0**	**Score 1**	**Score 2**	**Score 3**

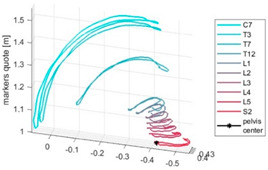
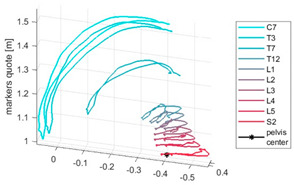
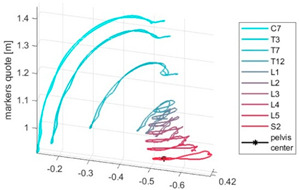
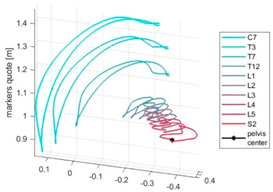
			

**Table A2:** Lateral Bending features.

Feature	Scoring Visual Guide			
**Fluency**	**Score 0** = the graph appears with fluid and continuous lines and curves.			
	**Score 0**	**Score 1**	**Score 2**	**Score 3**

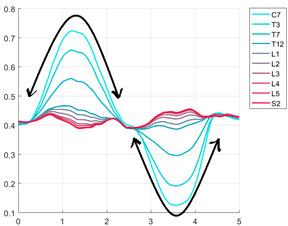
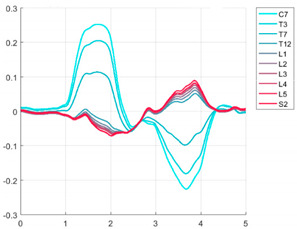
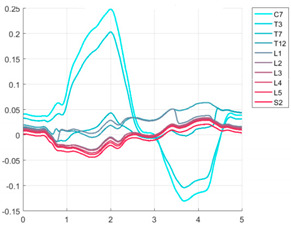
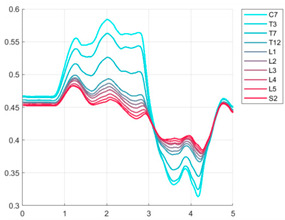
			
**Total** **Displacement** /**Range of Motion**	The displacement of segments trajectories			
	**Score 0**	**Score 1**	**Score 2**	**Score 3**

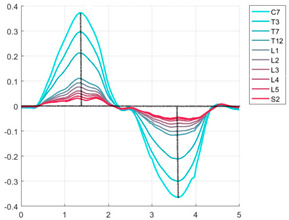
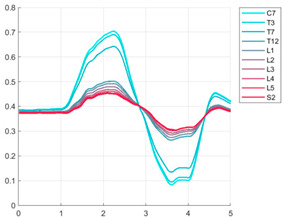
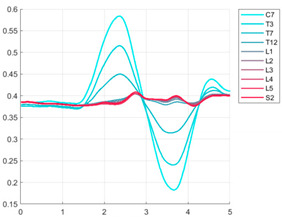
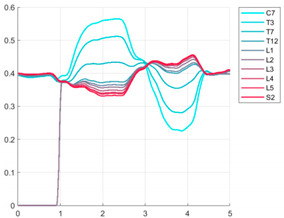
			
**Symmetry**	**Score 0** = the descending part of the graph overlaps with the ascending part			
	**Score 0**	**Score 1**	**Score 2**	**Score 3**

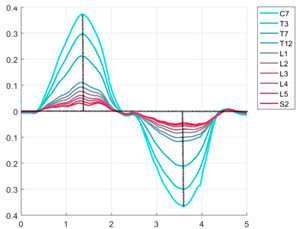
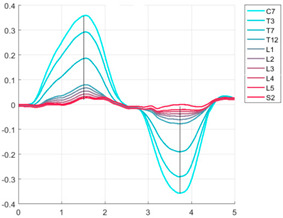
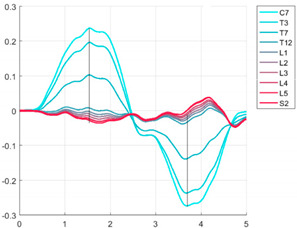
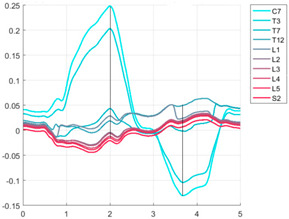
			
**Cervico-Thoracic/lu** **Mbosacral** **Reverse** **Movement**	**Score 0** = when observing the movements of C7 and S2, their movements should be in an opposite trend with obviously a different magnitude.			
	**Score 0**	**Score 1**	**Score 2**	**Score 3**

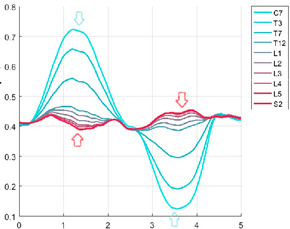
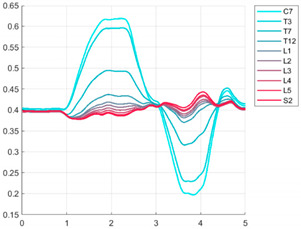
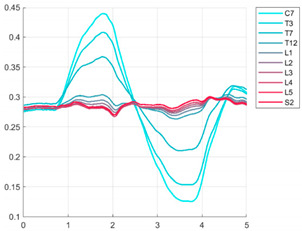
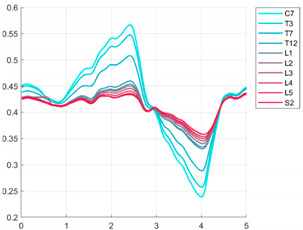
			
**Pelvis Centre Movement**	**Score 0** = the pelvis centre should be aligned with the midpoint of each line of the graph.			
	**Score 0**	**Score 1**	**Score 2**	**Score 3**

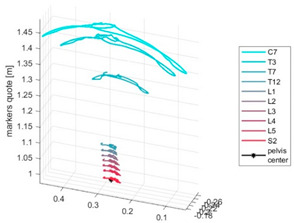
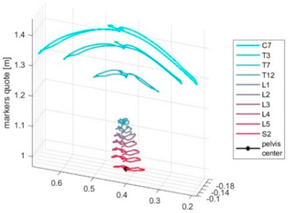
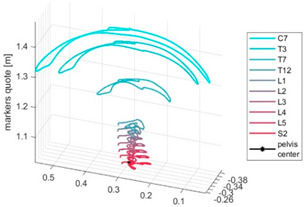
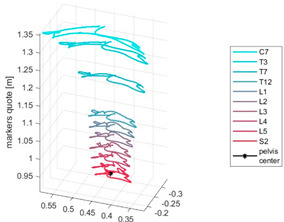
			

**Table A3:** Rotation features.

Feature	Description			
**Fluency**	**Score 0** = the graph lines are continuous with no intermittences.			
	**Score 0**	**Score 1**	**Score 2**	**Score 3**

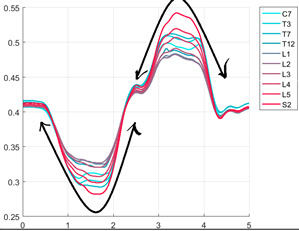
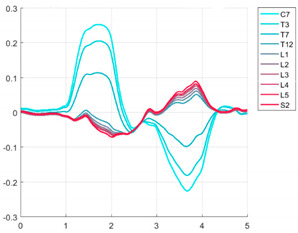
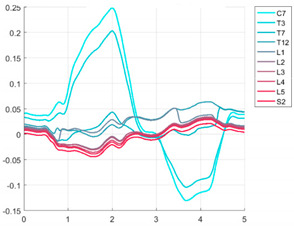
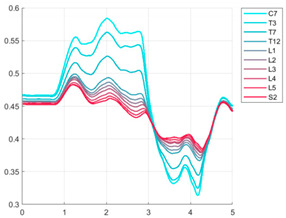
			
**Total** **Displacement/** **Range** **of Motion**	The displacement of segments trajectories			
	**Score 0**	**Score 1**	**Score 2**	**Score 3**

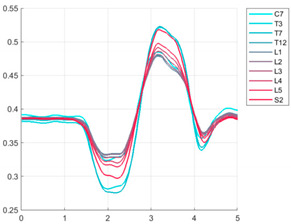
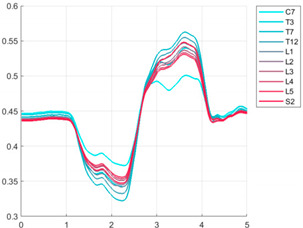
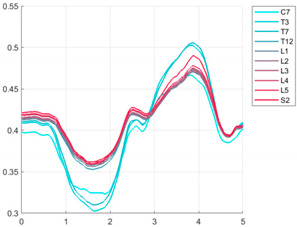
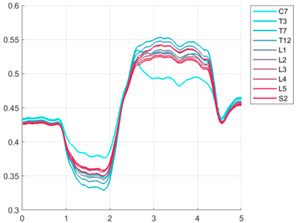
			
**Symmetry**	**Score 0** = dividing graph at the maximum and minimum point, the two parts are overlapping.			
	**Score 0**	**Score 1**	**Score 2**	**Score 3**

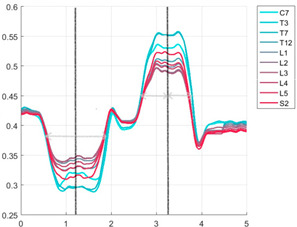
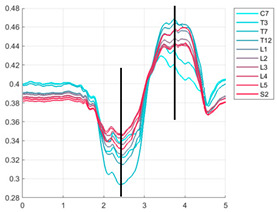
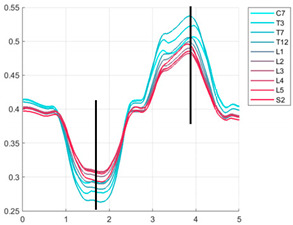
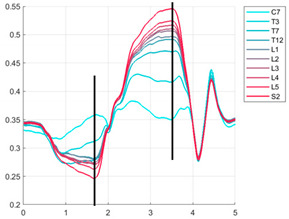
			
**End**	**Score 0** = at the end of the movement, the S2 line arrives at the same level as the start.			
	**Score 0**	**Score 1**	**Score 2**	**Score 3**

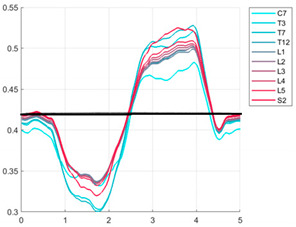
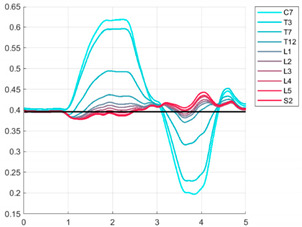
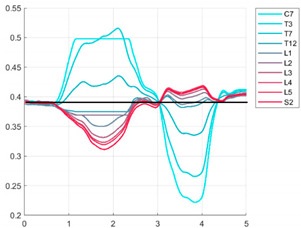
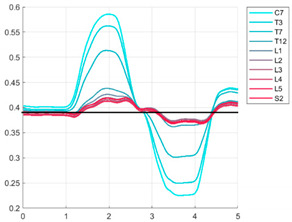
			
**Pelvis Centre Movement**	**Score 0** = pelvis centre should be aligned with the midpoint of each line of the graph.			
	**Score 0**	**Score 1**	**Score 2**	**Score 3**
			
